# Identification of rare variants causing urea cycle disorders: A clinical, genetic, and biophysical study

**DOI:** 10.1111/jcmm.16379

**Published:** 2021-02-21

**Authors:** Fang Liu, Li‐sha Bao, Ru‐jia Liang, Xiao‐ying Zhao, Zhi Li, Zhi‐fang Du, Shao‐guang Lv

**Affiliations:** ^1^ Department of Pediatrics NICU Bethune International Peace Hospital (the 980th Hospital of the People's Liberation Army Joint Service Support Force) Shijiazhuang China

**Keywords:** argininosuccinic aciduria, carbamoyl phosphate synthetase 1deficiency, citrin deficiency, ornithine transcarbamylase deficiency, urea cycle disorder, whole‐exome sequencing

## Abstract

Urea cycle disorders (UCDs) are a group of rare metabolic conditions characterized by hyperammonemia and a broad spectrum of phenotypic severity. They are caused by the congenital deficiency in the eight biomolecules involved in urea cycle. In the present study, five cases of UCD were recruited and submitted to a series of clinical, biochemical, and genetic analysis with a combination of high throughput techniques. Moreover, in silico analysis was conducted on the identified missense genetic variants. Various clinical and biochemical indications (including profiles of amino acids and urinary orotic acids) of UCD were manifested by the five probands. Sequence analysis revealed nine diagnostic variants, including three novel ones, which caused Argininosuccinic aciduria (ASA) in one case, Carbamoyl phosphate synthetase 1deficiency (CPS1D) in two cases, Ornithine transcarbamylase deficiency (OTCD) in one case, and Citrin deficiency in 1case. Results of i*n silico* biophysical analysis strongly suggested the pathogenicity of each the five missense variants and provided insight into their intramolecular impacts. In conclusion, this study expanded the genetic variation spectrum of UCD, gave solid evidence for counselling to the affected families, and should facilitate the functional study on the proteins in urea cycle.

## 1 INTRODUCTION

The urea cycle (UC) is a metabolic pathway for the disposal of excess nitrogen, which arises primarily as ammonia.^1^ Urea cycle disorders (UCDs), presenting with hyperammonemia that arise in either the neonatal period or later with an estimated compound incidence of ~ 1:35,000 and a high mortality (25%‐50%),^2^, ^3^ are a category of rare inherited metabolic conditions that impair the effectiveness of UC due to the congenital defects of enzymes or transporters in the UC.^4^ The common feature of UCDs is hyperammonemia, but the severity vary greatly because the variations causing the disease may be associated with deficiencies in at least eight proteins at different stages of UC and may damage them to varying degrees.^5^, ^6^ These eight proteins include six enzymes, namely N‐acetylglutamate synthase (NAGS, EC 2.3.1.1), carbamoyl phosphate synthase I (CPS1, EC 6.3.4.16), ornithine transcarbamylase (OTC, EC 2.1.3.3), argininosuccinate synthase (ASS, EC 6.3.4.5), argininosuccinate lyase (ASL, EC 4.3.2.1) and arginase (ARG, EC 3.5.3.1), and two amino acid transporters, namely ornithine transporter (ORNT1; ornithine/citrulline carrier) and citrin (aspartate/glutamate carrier).^5^, ^6^ Generally speaking, hyperammonemia leads to anorexia, cerebral oedema, lethargy, vomiting, hypothermia, hyperventilation (or hypoventilation), neurologic posturing, and coma.^6^ The ability to accurately identify the pathogenesis in early stage of the disease is beneficial for taking appropriate and necessary management to prevent irreversible neurological damage.^7^, ^8^

Plasma ammonia level is the most direct indicator of UCD patients at any age, yet it does not meet the requirement for screening for early treatment. Currently, tandem MS of dried blood spots is the widely used method to screen for neonatal metabolic diseases, including UCDs.^9^ However, the sensitivity and specificity of this method for detection of reducing citrulline are not high, resulting in a decrease in the detection rate of some types of UCDs, such as NAGS, CPS1,and OTC deficiencies.^10^ The characteristics of clinical, genetic heterogeneity and variable severity of UCDs promoted the development of more comprehensive and individualized diagnostic and therapeutic strategies.^11^ In addition, identification of the proband's genotype is of great importance in the selection of reproductive options, such as prenatal diagnosis (PD) or pre‐implantation genetic diagnosis (PGD), for affected families. Enzyme activity analysis, although being a time/labour consuming assay, can help confirming the diagnosis and the residual activity if the genetic test does not give a clear result,^6^ so it should be retained.

In the present study, 5 families with early onset metabolite disorders were recruited and underwent a series of clinical, biochemical and genetic tests. The results provided clinicians with sufficient evidence to develop specific treatment measures. Furthermore, structural prediction and molecular dynamic analysis were conducted to elucidate the intramolecular impact of missense variants.

## 2 MATERIALS AND METHODS

### 2.1 Subjects

This project was approved by the Ethics Committee of the Bethune International Peace Hospital (the 980th Hospital of the People's Liberation Army Joint Service Support Force) (Approval No. 20 180 023). The parents of these patients gave written informed consent for clinical examination, genetic analysis and data publication.

**FIGURE 1:**
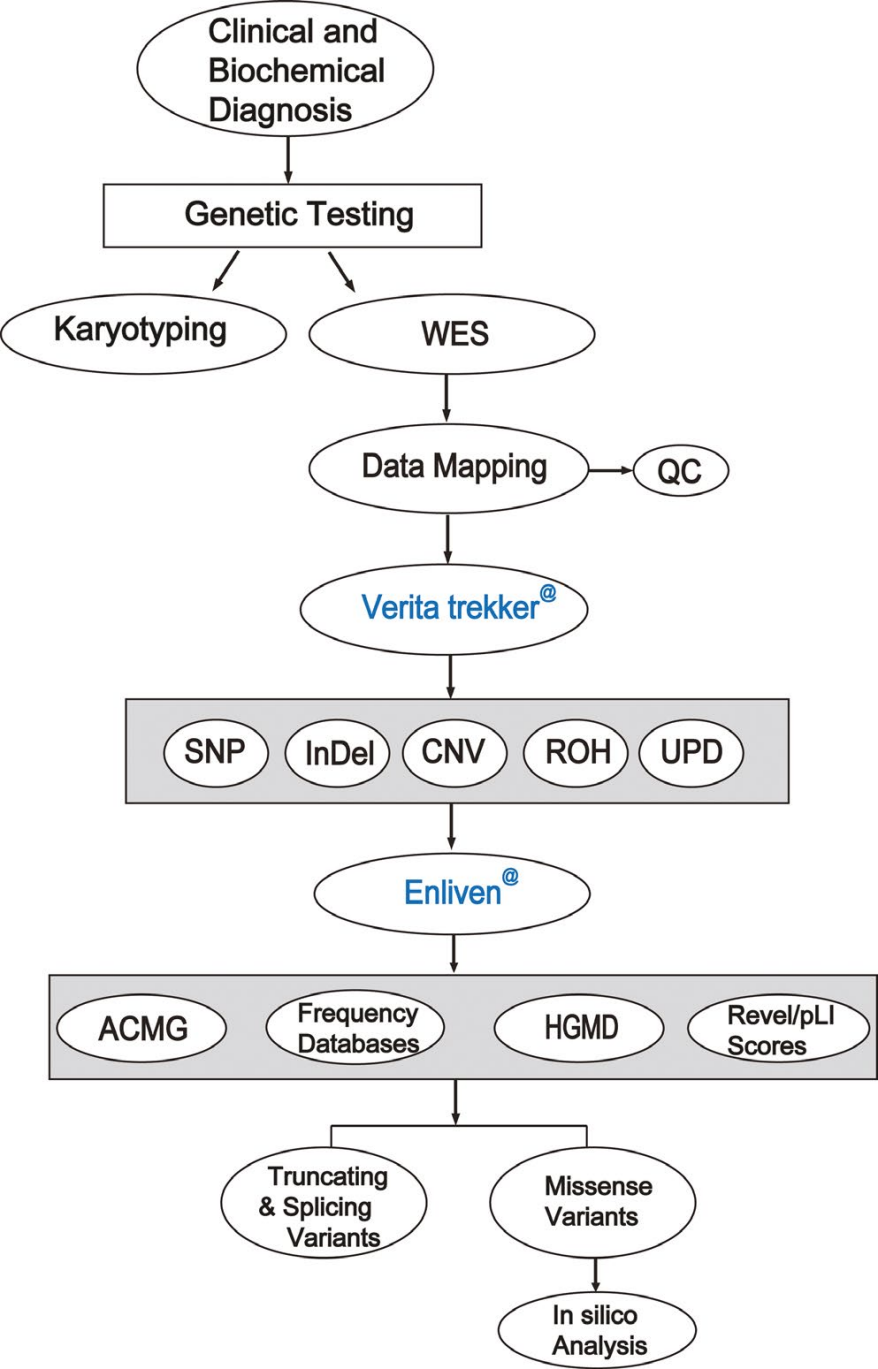
The overall experimental workflow in this study. ACMG, American College of Medical Genetics and Genomics; Aqua words represent patented software of Berry Genomics; CNV, copy number variation; HGMD, Human Gene Mutation Database; InDel, insertion/deletion; Mt, mitochondrial variation; QC, quality control; ROH, runs of homozygosity; SNP, single nucleotide polymorphism; WES, whole‐exome sequencing

Five families with suspected metabolic disorders were recruited in the Department of Pediatrics of Bethune International Peace Hospital between July 2018 and March 2020. The overall study workflow was shown in Figure 1.

### 2.2 Clinical and biochemical analysis

Probands of these families received comprehensive clinical examination and laboratory tests. The diagnostic criteria for UCD referred to that summarized by Matsumoto et al.^6^ General biomedical tests were performed using Cobas C501 Automatic System (Roche, Switzerland). Tests of coagulation function were carried out using ACL TOP 700LAS System (Instrumentation Laboratory, Werfen, Spain). The plasma amino acid profiles were tested using API 3200MD LC‐MS/MS tandem mass spectrometry System (AB SCIEX, Canada), and the urinary orotic acid levels were tested using GC‐2010 Pro Capillary Gas Chromatograph System (Shimadzu, Japan).

Specific management strategy was adopted to treat each proband according to the combined results of clinical, biomedical and genetic analysis.

### 2.3 Genetic analysis

Conventional G‐bangding karyotyping was performed on peripheral blood specimens from the 5 probands according to the AGT (American Genetic Technician) cytogenetics manual,^12^ to detect overall chromosomal anomalies.

Whole‐exome sequencing (WES) was conducted on samples of the 5 probands as described in previous studies.^13^, ^14^ Genomic DNA (1 µg) was extracted from 200 µl peripheral blood using the DNA Blood Midi/Mini kit (Qiagen GmbH), which then underwent quality control using agarose gel electrophoresis and UV spectrophotometry. DNA fragments were hybridized and captured by xGen.

Exome Research Panel (Integrated DNA Technologies, Inc) according to the manufacturer's protocol. The libraries were tested for enrichment by quantitative PCR, and the size, distribution and concentration were determined using an Agilent.

Bioanalyzer 2100 (Agilent Technologies, Inc). The NovaSeq 6000 platform (Illumina, Inc), along with ~ 150 bp pair‐end reads, was used for the genomic sequencing of DNA with ~ 300 pM per sample using NovaSeq Reagent kit. Sequencing raw reads (quality level %Q30 > 89%) were aligned to the human reference genome (accession no. hg19/GRCh37; http://hgdownload.cse.ucsc.edu/golden‐Path/hg19/chromosomes/) using the Burrows‑Wheeler Aligner tool and the PCR duplicates were removed using Picard v1.57 (http://picard.sourceforge.net/). Variant calling was performed with the Verita Trekker^®^ Variants Detection system (v2.0; Berry Genomics, Inc) and Genome Analysis Toolkit (https://software.broadinstitute.org/gatk/). Variants were then annotated and interpreted using ANNOVAR (v2.0) and Enliven^®^ Variants Annotation Interpretation systems (Berry Genomics, Inc),^15^ based on the common guidelines by American College of Medical Genetics and Genomics (ACMG).^16^ To assist in the interpretation of pathogenicity, we referred to 3 population frequency databases (1000G_2015aug_eas, https://www.internationalgenome.org; ExAC_EAS, http:// exac.broadinstitute.org; gnomAD_exome_EAS (http:// gnomad.broadinstitute.org) and HGMD (Human Gene Mutation Database) proV2019. Revel score^17^ (a combined method of pathogenicity prediction) and pLI score (representing the tolerance for truncating variants) were also introduced.

To validate variants, Sanger sequencing was introduced as a confirmatory method using 3500DX Genetic Analyzer (Applied Biosystems, Thermo Fisher Scientific, USA). The sequencing PCR primers, reaction conditions and reagents were included in Table S1.

### 2.4 In silico protein structural and molecular dynamic analysis

The evolutionary conservatism of all amino acids affected by missense variants was analysed using MEGA7 (http://www.megasoftware.net/previousVersions.php) with default parameters.

Protein modelling was conducted using Modeller 9V17 software^18^ with default parameters based on the structure models of homozygous sequences indexed in PDB database (http://www.rcsb.org/). Molecular dynamic analysis was conducted on 1 novel missense variant. Briefly, both wild‐type and mutant models were generated by Modeller 9V17; the programme CHARMM22 was used to add hydrogen atoms, N‐ and C‐terminal patches to the models.^19^ The models were solvated and neutralized in a box with TIP3P water at a minimum of 13 Å between the model and the wall of the box. All simulations were run using NAMD 2.9 with periodic boundary conditions (PBC) applied.^20^ The temperature was held at 300 K while the pressure was controlled at 1 atm. The time step was set to 2 fs and the particle mesh Ewald method was applied to model the electrostatics and the van der Waals interactions cutoff was set at 12 Å. Both models followed a three‐step pre‐equilibration totalling 600 ps, the last snapshots of which were chosen as the starting structures for 20 ns productive simulations without constraints.

## 3 RESULTS

### 3.1 Clinical findings

**FIGURE 2:**
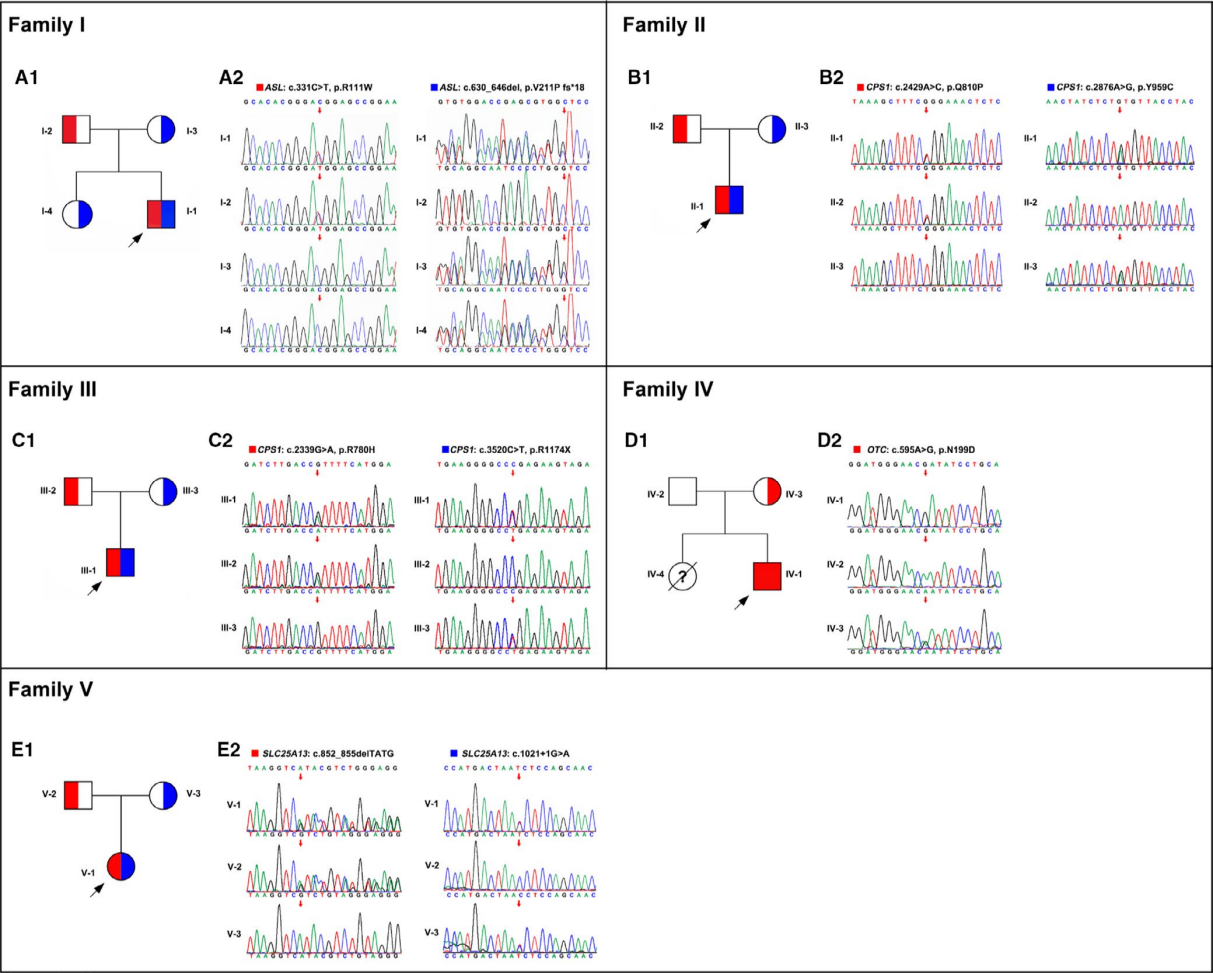
Pedigree diagrams and Sanger sequencing results of the five families. A1, B1, C1, D1 and E1, Pedigree diagrams and the variant carrying status in the five families. A2, B2, C2, D2 and E2, Sanger sequencing results of the diagnostic variants in the five families. The red/blue blocks represent corresponding variants

The pedigree diagrams of the 5 families were demonstrated in Figure 2.The detailed clinical indications of the 5 probands were summarized in Table 1, and the brief medical histories are as follows. Detailed treatment course and highlighted remarks were included in Table S1.

**TABLE 1:** Clinical and biochemical manifestations of the 5 probands

**Patient No**.	**Gender**	**Clinical findings and medical history**	**Laboratory Indications**		**AA, UOC Indications**	
			**Results**	**Reference**	**Results**	**Reference**
I‐1	M	Poor response, sucking rejection and hypotonia at 12th day after birth.	NH_3_: 200‐500 μmol/L ALT: 185.71 U/L AST: 78.30 U/L PT: 114 s INR: 10.27 Fib: 0.28 g/L TT: 36 s AT‐III: 3%	9‐30μmol/L 0‐40 U/L 6‐37 U/L 9.4‐12.5 s 0.8‐1.2 2.38‐4.98 g/L 10.3‐16.6 s 83%‐128%	**AA** Citrulline: 217.03 μmol/L Valine: 39.67μmol/L Ornithine/Citrulline: 0.14 Glutamate/Citrulline: 0.68 Citrulline/Phenylalanine: 8.23 **UOC** Arginosuccinate 1.8275 Creatine: 5.562 m/m C Guanidinoacetic acid: 0.008 m/m C	5.00‐30.00 μmol/L 50.00‐280.00 μmol/L 0.50‐6.50 1.50‐30.00 0.10‐1.20 / 0.004‐1.008 m/m C 0.011‐0.479 m/m C
II‐1	M	Poor response, sucking rejection at 4th day after birth.	NH_3_: 336 μmol/L	9‐30μmol/L	**AA** Citrulline: 3.01 μmol/L Methionine: 7.50 μmol/L Phenylalanine: 19.91μmol/L Tyrosine: 17.39 μmol/L Leucine: 56.35μmol/L **UOC** 3‐hydroxybutyric acid‐2:23.3μmol/L 4‐hydroxyphenyllactic acid‐2:66.9 μmol/L	5.00‐30.00 μmol/L 8.50‐45.00 μmol/L 25.00‐120.00 μmol/L 25.00‐250.00μmol/L 60.00‐25.00 μmol/L 0.0‐9.0 μmol/L 0.0‐20.0 μmol/L
III‐1	M	Poor response and hypotonia at 2nd day after birth.	NH_3_: 300‐900 μmol/L	9‐30μmol/L	**AA** Citrulline: 3.97 μmol/L Methionine: 5.67 μmol/L Phenylalanine: 16.54 μmol/L Tyrosine: 15.89 μmol/L Leucine: 49.45 μmol/L **UOC** 3‐hydroxybutyric acid‐2:37.4 μmol/L 4‐hydroxyphenyllactic acid‐2:70.6	5.00‐30.00 μmol/L 8.50‐45.00 μmol/L 25.00‐120.00 μmol/L 25.00‐250.00μmol/L 60.00‐25.00 μmol/L 0.0‐9.0 μmol/L 0.0‐20.0
IV‐1	M	Hyperammonemia right after birth; a elder sister as suspected patient with metabolic disease who died at 3 yr old.	NH_3_: 180 μmol/L (4 hours); 428‐3000μmol/L (inpatient)	9‐30μmol/L	**AA** Citrulline: 4.50 μmol/L Methionine: 50.51μmol/L Ornithine: 97.52μmol/L Glutamine: 110.88μmol/L **UOC** Lactic acid: 18.8μmol/L Pyruvate‐OX‐2:90.1 μmol/L 3‐hydroxybutyric acid‐2:33.2 μmol/L 4‐hydroxyphenyllactic acid‐2:110.4 μmol/L	5.00‐30.00 μmol/L 8.50‐45.00 μmol/L 9.00‐85.00 μmol/L 1.05‐30.00 μmol/L 0.0‐13.0 μmol/L 0.0‐30.0 μmol/L 0.0‐9.0 μmol/L 0.0‐20.0 μmol/L
V‐1	F	Intrauterine hypoxia; low‐birth weight; jaundice (35 days after birth)	ALT: 58 U/L D‐Bil: 5.70 μmol/L I‐Bil: 174.20 μmol/L AFP: 1200 ng/mL	0‐40 U/L 1.00‐2.50 μmol/L 0‐17.10 μmol/L <7 ng/mL	**AA** Citrulline: 451.17 μmol/L Threonine: 227.93 μmol/L Ornithine/Citrulline: 0.18 Glutamate/Citrulline: 0.20 Citrulline/Phenylalanine: 12.47 Threonine/Phenylalanine: 6.30 **UOC** 4‐hydroxyphenyllactic acid‐2:749.8 μmol/L 4‐ hydroxyphenylphruvic acid‐OX‐2:92.7 μmol/L	5.00‐30.00 μmol/L 15.00‐150.00 μmol/L 0.50‐6.50 1.50‐30.00 0.10‐1.20 0.20‐4.00 0‐20.0 μmol/L 0‐5.0 μmol/L

Abbreviations: AA, amino acid; AFP, Alphafetoprotein; ALT, alanine aminotransferase; AST, aspartate aminotransferase; AT‐III, antithrombin‐III; D‐Bil, Direct Bilirubin; F, Female; Fib, fibrinogen content; I‐Bil, Indirect Bilirubin; INR, international normalization ratio; M, Male; m/m C, mmol/mmol Creatinine; NH_3_, blood ammonia; PT, prothrombin time; TT, thrombin time; UOC, urinary orotic acid.

Patient I‐1, male, was hospitalized for poor response, sucking rejection and hypotonia at 12 days of age. Laboratory tests suggested hyperammonemia, hepatic dysfunction, dysfunction of blood coagulation and abnormal profiles of amino acids (AA) and urinary orotic acids (UOA) (Table 1). The peak area of the arginosuccinate was 1.8275 (677 times of the control level). The patient was treated with ornithine, aspartic acid and arginine injection and the ammonia level decreased to 286 μmol/L after 12 hours, and to 64 μmol/L 3 days later. Compound glycyrrhizin and reduced glutathione were used for protecting liver function. After treatment for 28 days, the ammonia level decreased to 54 μmol/L. Then the patient received a liver transplantation (LT) at 6 months. The surgery was successful and the infant is currently in good condition.

Patient II‐1, male, was hospitalized for poor response, sucking rejection at 4 days of age. Laboratory tests suggested hyperammonemia, yet normal hepatic function and blood glucose with no metabolic acidosis. The abnormal AA and UOA profiles were demonstrated in Table 1. The infant was treated with arginine hydrochloride injection and the ammonia level had been reduced to 84 μmol/L. But with a tiny amount of protein intake, his blood ammonia was elevated (like taking 30ml milk, once every 3 hours, then the blood ammonia would increase to ~ 300 μmol/L, with no effectiveness by taking oral anti‐hyperammonia drugs such as sodium benzoate). The parents rejected LT. At the 53th day, his condition deteriorated (ammonia level > 500 μmol/L) and developed into death.

Patient III‐1, male, was hospitalized for poor response and hypotonia at 2 days after birth, and developed into convulsions and coma during hospitalization. Laboratory tests indicated severe hyperammonemia and abnormal AA/UOA profiles (Table 1). After 3 weeks’ dietary and medicinal treatment, the infant's ammonia level dropped to normal, but a cranial magnetic resonance imaging scan revealed abnormity. He was diagnosed with cerebral palsy at 1 years old.

Patient IV‐1, male, having a suspected family history (details in Table 1), was hospitalized for hyperammonemia right after birth. Shortly after admission, the patient developed pulmonary bleeding, upper gastrointestinal bleeding, convulsions and coma. Despite of aggressive emergency treatment, the child died 24 hours after birth.

Patient V‐1, female, was hospitalized for jaundice at 35 days after birth. She was once with *in utero* foetal hypoxia, and her birth weight was 2100g with 45cm of birth length (<3rd percentile, both). Laboratory results indicated her with liver dysfunction (probably intrahepatic cholestasis) and irregular AA/UOA profile (Table 1). After a thorough genetic test, she was given treatment with lactose‐free milk powder and ursodeoxycholic acid. Her hepatic function returned to normal at 3 months old; and the AFP level decreased gradually, returning to normal by 1 year old.

### 3.2 Genetic variations

The karyotyping results were normal for each of the probands.

By WES, a total of 10 diagnostic variants were detected from the 6 families, involving with 4 genes, which were *ASL* (MIM *608 310), *CPS1* (MIM *608 307), *OTC* (MIM *300 461), and *SLC25A13* (MIM *603 859). Carrier status is shown in Table 2 and Figure 2. Among them, three variants, namely *ASL*: c.630_646del (p. V211P fs*18), *CPS1*: c.2429A > C (p. Q810P), and *SLC25A13*: c.1021 + 1G>A, were novelly identified.

**TABLE 2:** Data of diagnostic variations detected in this study

**Pedigree No**.	**Disorder (MIM No.)**	**Variant No**.	**Carriers ID (in Figure ** **1** **)**	**Gene (MIM No.); Transcript No**.	**cDNA alteration**	**Protein alteration**	**Frequency in 3 databases** ^a^	**NMD prediction**	**Revel score** ^b^	**pLI** ^c^	**HGMD** ^d^	**PMID** ^e^	**Level (Evidence)** ^f^
I	ASA (#207900)	1	I‐1; I‐2	*ASL* (*608 310); NM_000048	Exon5: c.331C > T	p. R111W	0;0;0	NO	0.89	3.85E‐08	DM	1 705 937	LP(PS1, PM2, PM1, PP3)
		2	I‐1; I‐3; I‐4		Exon9: c.631_647del	p. V211Pfs*18	0;0;0	YES,0.49	‐	3.85E‐08	‐	novel	P(PVS1, PM2, PM3)
II	CPS1D (#237300)	3	II‐1; II‐2	*CPS1* (*608 307); NM_001875	Exon20: c.2429A > C	p. Q810P	0;0;0	NO	0.97	0.0833574	‐	novel	VUS(PM2, PM5, PP3)
		4	II‐1; II‐3		Exon23: c.2876A > G	p. Y959C	0;0;0	NO	0.99	0.0833574	DM	21 120 950	LP(PS1, PM2, PP3)
III	CPS1D (#237300)	5	III‐1; III‐2	*CPS1* (*608 307); NM_001875	Exon19: c.2339G > A	p. R780H	0;0;0	NO	0.863	0.0833574	DM	17 310 273; 27 436 290; 28 526 534	VUS(PM1, PM2, PP3)
		6	III‐1; III‐3		Exon29: c.3520C > T	p. R1174X	0;0;0	YES,0.78	‐	0.0833574	DM	21 120 950	LP(PVS1, PM2)
IV	OTCD (#311250)	7	IV‐1; IV‐3	*OTC* (*300 461); NM_000531	Exon6: c.595A > G	p. N199D	0;0;0	NO	0.938	0.9810179	DM	11 793 468	P(PS4, PM1, PM2, PM5, PP3, PP4)
V	NICCD (#605814)	8	V‐1; V‐2	*SLC25A13*(*603 859); NM_001160210	Exon9: c.852_855delTATG	p. M285Pfs*2	0;0;0	YES,0.42	‐	1.94E‐15	‐	10 369 257	LP(PVS1, PM2)
		9	V‐1; V‐3		Exon10: c.1021 + 1G>A	/	0;0;0	YES,0.55	‐	1.94E‐15	DM	novel	LP(PVS1, PM2)

Abbreviation: NMD, nonsense‐mediated RNA decay.

^a^ 1000g2015aug_eas(https://www.internationalgenome.org/); ExAC_EAS(http://exac.broadinstitute.org); gnomAD_exome_EAS(http://gnomad.broadinstitute.org/);

^b^ Revel score, an ensemble method for predicting the pathogenicity of missense variants on the basis of individual tools: MutPred, FATHMM, VEST, PolyPhen, SIFT, PROVEAN, MutationAssessor, MutationTaster, LRT, GERP, SiPhy, phyloP, and phastCons (http://dx.doi.org/10.1016/j.ajhg.2016.08.016);

^c^ pLI: https://gnomad.broadinstitute.org/;

^d^ HGMD^®^: Human Gene Mutation Database (Professional Version 2019.4);

^e^ PMID: PubMed ID(https://pubmed.ncbi.nlm.nih.gov/);

^f^ ACMG: The American College of Medical Genetics and Genomics; P: Pathogenic; LP: Likely Pathogenic; VUS: Variants of Unknown Significance; LB: Likely Benign; B: Benign.

Patient I‐1 carried a compound heterozygous variation consisting of *ASL*: c.331C > T and c.630_646del, which supported the diagnosis as argininosuccinic aciduria (ASA, MIM #207900). The two variants were inherited from his parent, respectively, and the latter one was carried by his elder sister (I.4) also. Patient II‐1 and III‐1 were both diagnosed as carbamoyl phosphate synthetase 1deficiency (CPS1D, MIM #237300) since they both carried compound heterozygous variations in *CPS1* gene, which are c.2429A > C/ c.2876A > G (in Patient II‐1) and c.2339G > A/ c.3520C > T (in Patient III‐1), inherited from their parents, respectively. Patient IV‐1 was affected by the X‐link ornithine transcarbamylase deficiency (OTCD, MIM #311250) since he carried a known pathogenic variant, *OTC*: c.595A > G inherited from his asymptomatic heterozygous mother (IV.3). Patient V‐1 was affected by citrullinemia type II, neonate‐onset (aka NICCD, MIM #605814), caused by the compound heterozygous variants in *SLC25A13* gene, *SLC25A13*: c.852_855delTATG/ c.1021 + 1G>A, inherited from the asymptomatic parents, respectively.

### 3.3 Structural and molecular dynamic analysis

**FIGURE 3:**
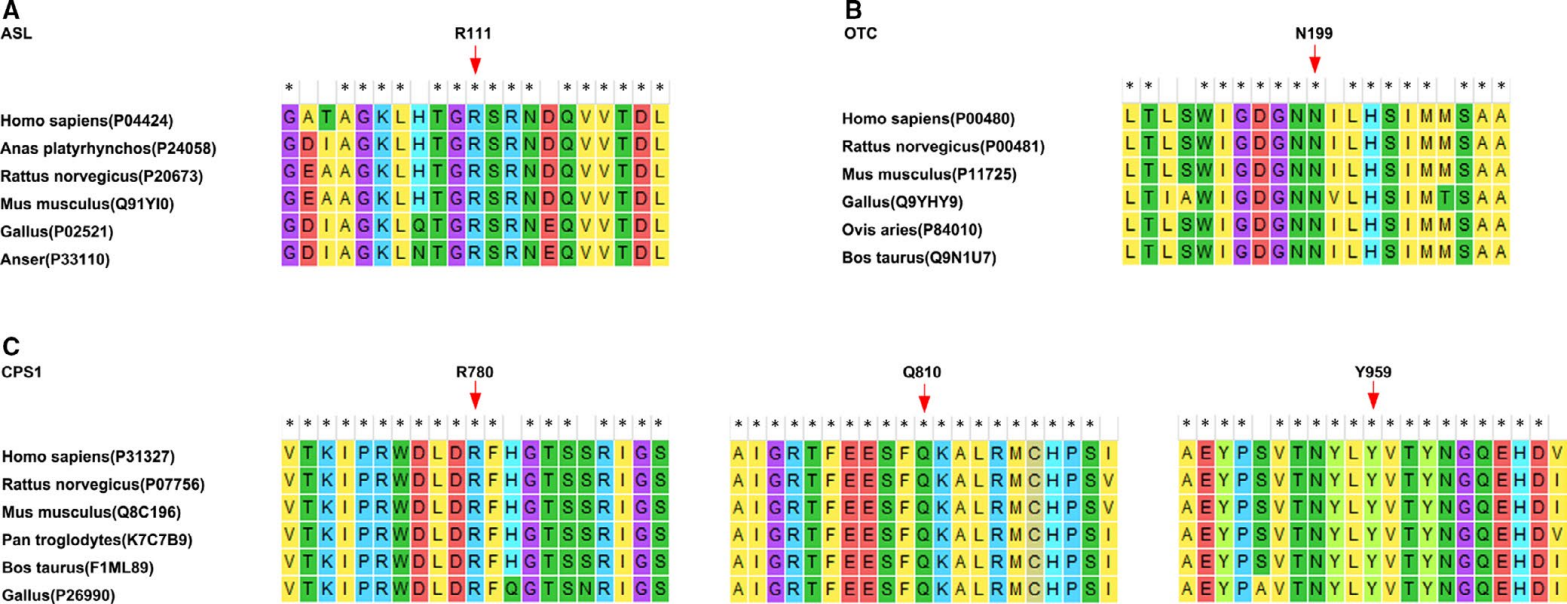
Conservatism status of the corresponding amino acids of the five missense variants detected in this study among species. A, the R^111^ residue in ASL protein; B, the N^199^ residue in OTC protein; C, the R^780^, Q^810^ and Y^959^ residues in CPS1 protein

In this study, 5 diagnostic missense variants were detected, namely *ASL*: c.311C > T(p. R111W), *OTC*: c.595A > G(p. N199D), *CPS1*: c.2429C > A(p. Q810P), *CPS1*: c.2876A > G(p. Y959C), and *CPS1*: c.2339G > A(p. R780H). The amino acids they altered maintained evolutionary conservation among species (Figure 3).

**FIGURE 4:**
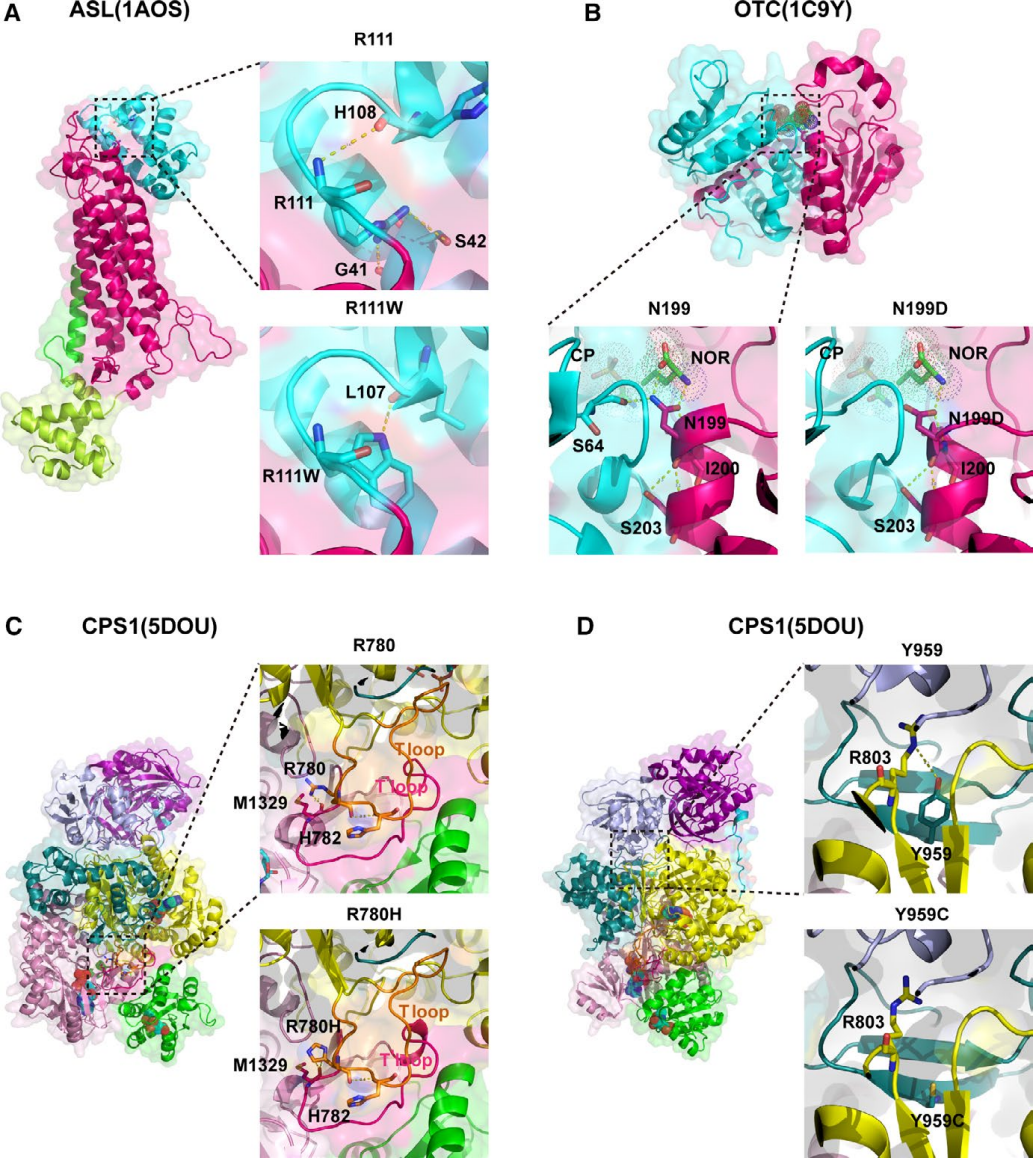
Structural prediction results of the four missense variants. Dotted yellow lines represent hydrogen bonds; N‐acetyl‐L‐glutamate (NAG) and two ADPs are shown in stick and dot representation. A, R^111^ and R111W models of ASL protein; B, N^199^ and N199D models of OTC protein, (NOR, L‐Norvaline; CP, carbamoyl phosphate); C, R^780^ and R780H models of CPS1 protein, D, Y959 and Y959C models of CPS1 protein

The analysis of *ASL*: c.311C > T(p. R111W) was based on the model PDB: 1AOS.^21^ The variant R111W replaced a strongly basic arginine by a large amino acid with benzene rings, thereby broke the hydrogen bonds formed by the side chain of R^111^and expectantly changed its electrostatic interactions. Consequently, it may change the conformation of the loop which the R^111^ belongs to (Figure 4A). *OTC*: c.595A > G(p. N199D) was analysed based on the model PDB: 1C9Y.^22^ The side chain of N^199^points to active site and forms 3 hydrogen bonds with NOR(L‐norvaline ) and S^64^. N199D replaced the polar asparagine with a strongly basic amino acid, thereby broke the hydrogen bonds formed by the side chain of N^199^and expectantly changed its electrostatic interactions and/or networks of hydrogen bondselectrostatic interactions and/or networks of hydrogen bonds. Consequently, it may change the conformation of the active site so as not to catalyse the formation of citrulline (Figure 4B). The other 3 variants were analysed based on the model PDB: 5DOU.^23^ R^780^ belongs to the T loop and forms a hydrogen bond with M^1329^. R780H replaced the strongly basic arginine by a large and polar amino acid, broke the interaction with the T' loop, and expectantly changed its electrostatic interactions and/or networks of hydrogen bonds. Consequently, it may change the conformation of the T loop and T^'^ loop (Figure 4C). Y^959^ belongs to the integrating domain that concatenates the two carbamate synthetase ATP domains. Y959C broke the interaction with the carbamate synthetase ATP A domain, which could change the topology between the integrating domain and the two carbamate synthetase ATP domains (Figure 4D).

**FIGURE 5:**
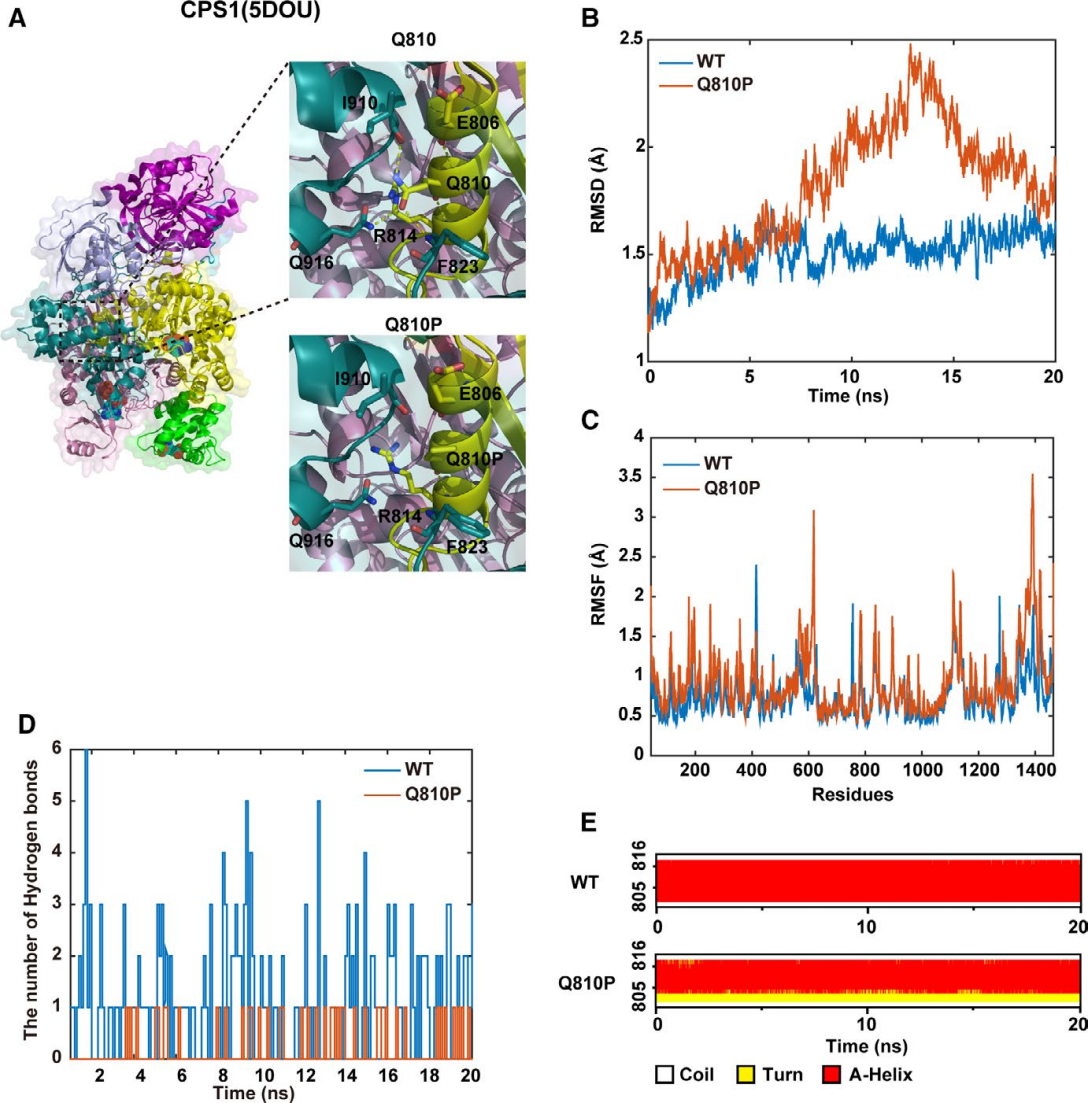
Results of the molecular dynamic analysis on CPS1: p. Q810P variant. A, The structure models of domains containing Q^810^and Q810P (Dotted yellow lines represent the hydrogen bonds; N‐acetyl‐L‐glutamate (NAG) and two ADPs are shown in stick and dot representation); B, The trajectory of RMSD (Cα) of the wild‐type (WT) and Q810P proteins; C, RMSF of the two proteins calculated from each simulation; D, Secondary structural components of corresponding region as a function of time; E, The number of hydrogen bonds formed between Q^810^ or Q810P and the rest residues in the two proteins, respectively

The variant *CPS1*: c.2429A > C (p. Q810P) identified from Patient II‐1was a novel missense variant with unknown clinical and biophysical significance. Structurally, Q^810^ is located in the middle of a helix. Q810P broke the four hydrogen bonds formed by Q^810^ (Figure 5A). P acts as a helix breaker because the rotation around the N‐Ca bond is impossible, so Q810P may change the secondary structure then change the whole conformation. It was also indicated that the Q810P is more flexible than the wild‐type (WT) according to the trajectory of RMSD (Root Mean Square Deviation) and RMSF (Root Mean Square Fluctuation) (Figure 5B,C). Besides, proline whose last atom of side chain bonds to the N atom of main chain prefers the first round of helices, but Q810P placed in the middle of a helix, which always causes an apparent bent (shown in Figure 5E). Moreover, the number of hydrogen bonds formed between Q^810^and the rest residues were more than that formed between Q810P and the rest residues (1.155:0.20) (Figure 5D).

## 4 DISCUSSION

Ammonia is produced by intestinal urease‐positive bacteria as well as during amino acid metabolism, and the urea cycle (UC) performs the critical function of converting ammonia into urea.^6^

In the current study, the 5 cases involved 4 various UCD types, respectively. ASA, resulted from ASL deficiency, is consistent with autosomal recessive (AR) pattern and may present a broad spectrum of severity depending on the residual activity of ASL.^24^, ^25^ Recently, a model was developed to predict the phenotypic severity of ASA patients by cohort based multi‐platform data, which is worth learning from, but also needs to be optimized by expanding the sample size.^26^ Another study proved that cerebral condition in ASA involves neuronal oxidative/nitrosative stress independent of hyperammonaemia, which should be highly valued in clinical practice.^27^ In the present study, patient I‐1 manifested significant noenatal elevation of plasma ammonia and citrulline, and the Arginosuccinate level was more than 600 times higher than the control; furthermore, the identification of compound variants in *ASL* gene confirmed the ASA diagnosis. The use of ornithine, aspartic acid and arginine injection combination therapy is an experimental attempt to suppress hyperammonia in the absence of hemodialysis equipment and Sodium benzoate in our unit, which does not necessarily suit all centres The follow‐up information suggested that the ammonia level and liver function returned to normal, and no trichorrhexis nodosa appeared after the LT operation. Yer, the practical effects of LT are still controversial, since it was reported that LT cannot protect neural complications in some types of UCD.

As the first and rate‐limiting enzyme of UC, CPS1 catalyses the condensation of ammonia and bicarbonate into carbamoyl phosphate in the mitochondrial matrix.^28^ CPS1D is one of the most severe types of UCD.^5^, ^29^ Even rescued from acute crisis, patients are chronically at risk for repeated bouts of hyperammonemia and should be paid constant attention to.^30^ While early metabolic management and medication can relieve some of the symptoms, LT surgery can lead to better outcome for patients,^31^, ^32^ even from heterozygous donors.^33^ Patient II‐1 and III‐1 in this study were both affected by CPS1D and showed similar clinical and biochemical manifestations, yet the unavailability of timely LT led to their unsatisfactory situation. The establishment of CPS1 protein structure model is beneficial for elucidating the pathogenic role of missense mutations.^34^ The CPS1: p. Q810 residue remains conserved across species, and the pathogenicity of a Q810R variant has been reported.^34^, ^35^ Results of structuralprediction and/or MD revealed the submolecular impact of the 3 missense variants, which led to a better understanding of the pathogenicity.

OTCD is the most common type of UCD.^2^ Patient IV‐1 in this study presented a catastrophical deterioration within 24 hours after birth, which gave us little chance to carry out rescue measures. Even after successful treatment of neonatal hyperammonemic coma, these noenatal‐onset OTCD infants could easily become hyperammonemic again despite appropriate treatment; they typically require liver transplant by age 6 months to improve quality of life.^36^ The effect of p. N199D of *OTC* gene on active site may be the cause of enzyme inactivation according to the results of structural prediction.

Citrin deficiency, exhibiting exceeding phenotypic heterogenicity, can manifest in newborns or infants as neonatal intrahepatic cholestasis caused by citrin deficiency(NICCD), in older children as failure to thrive and dyslipidemia caused by citrin deficiency(FTTDCD), and in adults as recurrent hyperammonemia with neuropsychiatric symptoms in citrullinemia type II(CTLN2).^37^ The symptoms of NICCD is generally not severe and resolved by age one year with appropriate treatment, although a few cases involved LT.^38^ In this study, the blood ammonia of patient V‐1 did not increase significantly, indicating a better control of situation. Yet, after recovery, care should also be taken to avoid CTLN2 symptoms caused by stressors, for example, alcohol and sugar intake, medication, and/or surgery in her adult life.^39^

With respect to the genetic counselling on future pregnancies of these affected family, what needs attention is that the siblings of I‐1, II‐1, III‐1 and V‐1 will have a 25% risk of being affected, so reproductive options such as prenatal diagnosis or pre‐implantation diagnosis (with in‐vitro fertilization, IVF) are recommended, which also applies to case IV.^40^, ^41^ As a matter of fact, families I, III and V have made it clear that IVF approach will be considered. It was also suggested that the future spouse of subject I.4 should check his *ASL* genotype before they consider a pregnancy, and they should be made aware that de novo mutations are more common in males. As for case IV, the risk of male siblings of patient IV‐1 will be as high as 50%; while the female siblings, if being heterozygous, will not be entirely safe either and should avoid strong stressors as much as possible.^36^

As BabySeq projects emerged, the field of neonatal screening for mendelian diseases may enter a new era and fundamentally improve the efficiency of screening.^42^, ^43^, ^44^ However, there are many details, such as the design of gene panels, the curation of variation interpretation criteria, the choice of screening methods, the scope of application of subjects, etc, which need to be discussed in a long process.^45^ With regard to disorders like UCDs with acute neonatal presentation, it may be more appropriate to perform pre‐pregnancy carrier screening using a meticulously ‐designed gene panel.

A limitation of this study is that the first‐line genetic detection method we use is WES, covering only the gene coding exonic region and the adjacent splicing regions. As for the intronic variants and copy number variants as long deletions, it is undetectable only by WES, which requires attention in larger sample studies. Additionally, although genetic analysis is a good way for precise diagnose, clinicians need possible diagnosis emergently. Hence, Screening and chemical diagnosis is still important compared to gene analysis in clinical stage because we need more cost and time. Moreover, the functional impact and genotype‐phenotype correlations of specific variants for UCD need further elucidation.

In summary, we identified the causative variants in five families with UCDs, including three novel variants, which provided solid evidence for genetic counselling. In addition, thorough in silico analysis would benefit the interpretation of variations.

## CONFLICT OF INTEREST

The authors confirm that there are no conflicts of interest.

## AUTHOR CONTRIBUTIONS

**Fang Liu:** Conceptualization (lead); Funding acquisition (lead); Resources (lead); Supervision (lead); Writing‐review & editing (lead). **Li‐sha Bao:** Data curation (equal); Formal analysis (equal); Investigation (equal); Resources (equal); Validation (equal); Writing‐original draft (equal). **Ru‐jia Liang:** Formal analysis (equal); Investigation (equal); Methodology (equal). **Xiao‐ying Zhao:** Investigation (equal); Methodology (equal); Resources (equal); Software (equal). **Zhi Li:** Investigation (equal); Methodology (equal); Software (equal); Validation (equal). **Zhi‐fang Du:** Data curation (equal); Formal analysis (equal); Investigation (equal). **Shao‐guang Lv:** Investigation (equal); Methodology (equal); Project administration (equal).

## Supporting information

Table S1

## Data Availability

Based on reasonable requirements, all raw data can be obtained from the corresponding author.
