# Supplementary material for: Identification of rare variants causing urea cycle disorders: A clinical, genetic, and biophysical study
Source: J Cell Mol Med. 2021 Feb 21;25(8):4099–109. doi: 10.1111/jcmm.16379 (PMC8051738; doi:10.1111/jcmm.16379)
Supplement: Supplementary file 1 — Table S1 [file JCMM-25-4099-s001.docx]

**Table S1 Primers and PCR reaction conditions**

| **Fragment** | **Primers** | **Sequence** | **Size** | **Tm** | **Experiment system** |
| --- | --- | --- | --- | --- | --- |
| ASL-exon5 | ASL-exon5-F | 5'- GTAGGTTGGCAGGGCTGATG-3' | 380bp | 60℃ | TAQ |
|  | ASL-exon5-R | 5'- CCGATCCACCATGGTCCTAA-3' |  |  |  |
| ASL-exon9 | ASL-exon9-F | 5'-TCAGCCTGACATGTGGGAAC-3' | 447bp | 60℃ | TAQ |
|  | ASL-exon9-R­ | 5'-GAAGGTTTCACCAGGAGCCA-3' |  |  |  |
| CPS1-exon20 | CPS1-exon20-F | 5'-CCATGGAGAAAGTGAGAGAGG-3' | 595bp | 60℃ | TAQ |
|  | CPS1-exon20-R | 5'-CAATTTGCCCTTAAATCCAGG-3' |  |  |  |
| CPS1-exon23 | CPS1-exon23-F | 5'-GGGTTTCCAGAGACTAATAGAGAAT-3' | 683bp | 60℃ | TAQ |
|  | CPS1-exon23-R | 5'-ATGCTCTACCCATGCACCAA-3' |  |  |  |
| CPS1-exon19 | CPS1-exon19-F | 5'- TGAAGGTTGAGCGTTGTAGCA -3' | 559bp | 60℃ | TAQ |
|  | CPS1-exon19-R | 5'- CCATGTGTTGATGGTATCCAGG-3' |  |  |  |
| CPS1-exon29 | CPS1-exon29-F | 5'- TTGAGTATTTTGCAAGTATTGCCCT-3' | 568bp | 60℃ | TAQ |
|  | CPS1-exon29-R | 5'- AACCAGGATTCGATTGGGAAC-3' |  |  |  |
| OTC-exon6 | OTC-exon6-F | 5'- GACATTTTACCACGTTTTTGGGAT-3' | 453bp | 60℃ | TAQ |
|  | OTC-exon6-R | 5'-ATGGCCTACAGCAGGGTATTC-3' |  |  |  |
| SLC25A13-exon9 | SLC25A13-exon9-F | 5'-GAGGGCAGCAATCAGGAGAA-3' | 305bp | 60℃ | TAQ |
|  | SLC25A13-exon9-R | 5'-GCAACTGCAAGTGGAACAGG-3' |  |  |  |
| SLC25A13-exon10 | SLC25A13-exon10-F | 5'-TCATGGATTTAGAACCCAATGAGTA-3' | 470bp | 60℃ | TAQ |
|  | SLC25A13-exon10-R | 5'-GCACATTGCTACAGCCCAAC-3' |  |  |  |

**PCR amplification conditions**

| **Experiment system** | **PCR conditions** | **PCR enzyme and buffer** |
| --- | --- | --- |
| TAQ | Initial step of 95°C for 5 min, followed by 30 cycles of 95°C for 30 sec, 60℃ for 30sec, and 72°C for 30 sec, with a final extension step at 72°C for 10 mins. | TAKARA Ex Taq® |
